# Identifying parental chromosomes and genomic rearrangements in animal hybrid complexes of species with small genome size using Genomic *In Situ* Hybridization (GISH)

**DOI:** 10.3897/CompCytogen.v6i3.3543

**Published:** 2012-09-14

**Authors:** Massimiliano Rampin, Ke Bi, James P. Bogart, Maria João Collares-Pereira

**Affiliations:** 1 Centro de Biologia Ambiental - Faculdade de Ciências - Universidade de Lisboa, Campo Grande - 1749-016 Lisboa, Portugal; 2 Department of Integrative Biology, University of Guelph, Guelph, Ontario (Canada); 3 Museum of Vertebrate Zoology, University of California, Berkeley, California (USA)

**Keywords:** Allopolyploids, GISH, Hybrids, C-value, fishes, *Squalius alburnoides* complex

## Abstract

Genomic *In Situ* Hybridization (GISH) is a powerful tool to identify and to quantify genomic constituents in allopolyploids, and is mainly based on hybridization of highly and moderate repetitive sequences. In animals, as opposed to plants, GISH has not been widely used in part because there are technical problems in obtaining informative results. Using the allopolyploid *Squalius alburnoides* Steindachner, 1866 fish complex as a model system, we succeeded in overcoming methodological constraints when dealing with parental species with a small genome size. This hybridogenetic complex has biotypes with different genome compositions and ploidy levels, but parental chromosomes are small, morphologically very similar and therefore cannot be distinguished by conventional cytogenetic approaches. Specimens have a small genome (C-value1.2 pg) with a low level of highly and moderate repetitive sequences, mainly located at pericentromeric chromosome regions. Since it is well known that probe annealing depends on probe concentration and hybridization time to obtain uniform hybridization signals along the chromosome arms, we progressively increased the amount of labeled probes from 100ng up to 1µg and the incubation time from overnight up to 5 days. We also made other smaller improvements. Results showed a clear enhancement of signals with respect to previous data, allowing an accurate and reproducible assignment of the parental genomes in both diploid and triploid fish.It was thus evidenced that high probes’ concentrations and long incubation time are the key to obtain, without extra image editing, uniform and reliable hybridization signals in metaphase chromosomes of animal hybrids from species with small genome size.

## Introduction

Genomic *In Situ* Hybridization (GISH) was developed by Schwarzacher et al. (1989) to identify parental chromosomes in *Hordeum chilense* Roemer et Schultes x *Secale africanum* Stapf hybrid plants where classical karyotyping and/or chromosome banding were unable to detect genomic (chromosomal) differences. This technique uses labeled total genomic DNA as probes to recognize a genome in chromosome preparations of hybrid individuals. It provides a straight and simple visual identification of parental chromosomes and genome organization (e.g. chromosomal rearrangements) in interspecific/intergeneric hybrids and allopolyploid species, as well as introgression, addition and substitution lines (see e.g. Jiang and Gill 1994, Jacobsen et al. 1995, Fujiwara et al. 1997, Sanchez-Moran et al. 1999, Bi and Bogart 2006).

GISH uses labeled total genomic DNA (gDNA) as the probe in *in situ* hybridization experiments together with sheared unlabeled whole genomic DNA, usually from the other parental species (but see Markova and Vyskot 2009) as blocking DNA. The blocking DNA serves as a DNA competitor to avoid the staining of both genomes by the probe DNA if the parents are closely related. GISH works primarily on hybridization of highly and moderate repetitive DNA sequences (Markova and Vyskot 2009 and included references). The required amount of blocking DNA is known to depend on the phylogenetic distance between the parental species and it can be reduced for distantly related parental species. Based on *Nicotiana* Linnaeus allopolyploids, Lim et al. (2007) established a parental species divergence from around 1MY to 5MY for obtaining reliable results by GISH. Thus it is possible to distinguish parental chromosomes in interspecific hybrids using GISH if the parental genomes are divergent and the hybridization is relatively recent (Markova and Vyskot 2009).

Hybridization and polyploidy are known to be common phenomena in plants. Recent genomic studies have revealed a higher occurrence of these events in animals than previously suspected (Gromicho and Collares-Pereira 2007, Mallet 2007, Mable et al. 2011). GISH has been a powerful tool for analyzing plant hybrids and polyploids (reviewed in Abbasi et al. 2010). However in vertebrates it has only been successfully used in unisexual salamanders (Bi and Bogart 2006, Bi et al. 2007a,b, 2008, 2009, 2010), in hybridogenetic frogs (Zalesna et al. 2011) and in a salmonid hybrid study (Fujiwara et al. 1997). There are two other GISH applications in fishes, but one only shows centromeric hybridization signals (Zhu and Gui 2007) and the other did not include any illustration of GISH results (Valente et al. 2009). The lack of more applications in animals may be explained by the difficulty in obtaining accurate and reproducible results due to technical problems related to genome size and distribution of genome-specific repetitive sequences, as suggested by Ali et al. (2004) for plants. The high quality GISH studies in vertebrates were performed in species complexes with large genomes (data from Gregory 2011): *Ambystoma* Tschudi, 1838 (C-values ranging from 21.85pg-80.70pg) and *Pelophylax esculentus* Linnaeus, 1758 (5.60pg-11.53pg). Also in the *Oncorhynchus masou* Brevoort, 1856 x *Oncorhynchus mykiss* Walbaum, 1792 hybrid form, parental species have C-values ranging from 2.07pg-3.29pg and 1.87pg-2.92pg respectively, what may be considered relatively high in comparative terms for teleosts (reported C-values range from 0.35pg up to 4.90pg, mean value = 1.16pg – in Gregory 2011).

We aimed to improve GISH methodology using the *Squalius alburnoides* Iberian complex, as a model system. This fish complex originated from interspecific hybridization between *Squalius pyrenaicus* Günther, 1868 (P genome) as the maternal ancestor and a missing *Anaecypris*-like species (A genome) paternal ancestor. The complex is composed of different biotypes and ploidies (2n=50, 3n=75 and 4n=100), which are produced by non-sexual and sexual modes of reproduction. In southern Portugal, specimens carrying PA, PAA, PPA and PPAA genomes are found, as well as nuclear non-hybrid males AA which are reconstituted within the complex (reviewed in Gromicho and Collares-Pereira 2007, Collares-Pereira and Coelho 2010). Diploid specimens of *Squalius alburnoides* have a relatively small genome (C-value=1.2 pg; Próspero and Collares-Pereira 2000) and a low amount of highly and moderate repetitive sequences, mainly located at pericentromeric chromosome regions. Herein we describe an optimized protocol for obtaining reliable and informative results in hybrid fishes even if parental species have morphologically similar chromosome sets, relatively small chromosomes and C-values, which might be applicable to other animal hybrid genomes.

## Materials and methods

### Fish samples, ploidy and genome composition screenings

Chromosome preparations were obtained from cellular suspensions preserved in fixative (3:1 methanol:acetic acid) at -20°C for about 6 years from five diploid (PA genome, 2n=50) and one triploid (PAA genome, 3n=75) *Squalius alburnoides* specimens. The fish were selected from the offspring of artificial crosses obtained using specimens captured at Guadiana river basin in 2001–2003. Several specimens with an unknown genomic composition were also collected by electrofishing in October 2010 at Almargem drainage. Both populations are in Southern Portugal. The best two cellular suspensions from this pool (of two distinct triploid females) were also used for GISH experiments. Probe and blocking DNA were obtained from nuclear non-hybrid specimens of *Squalius alburnoides* (AA genome) and allopatric specimens of *Squalius pyrenaicus* (PP genome), respectively.

Ploidy of the fish was determined by analysis of erythrocyte DNA content using a Coulter Epics XL cytometer, following the method described in Collares-Pereira and Moreira da Costa (1999). Genome composition of the old suspensions was assessed following Crespo-López et al. (2006), and biotypes of the new sampled specimens were identified using the method of Sousa-Santos et al. (2005).

### Chromosomes preparation

Metaphase chromosomes were obtained from fibroblast fin cultures according to the method of Rodrigues and Collares Pereira (1996) with small modifications.After checking the quantity and quality of metaphases, the best suspensions were used for GISH experiments. In order to improve spreading of the metaphase plates, the slides were placed on a wet, cold (+4°C) sponge and one or two drops of the suspension were released onto high quality superfrost precleaned glass slides (Cole – Parmer, Vernon Hills Illinois). This treatment increases the surface tension allowing better separation of the chromosomes (open plates). The quality of the chromosome spreads was evaluated by phase-contrast microscopy for subsequent experiments. Selection criteria were: high number of metaphase plates, well separated chromosomes with few or no overlapping chromosome arms and little cytoplasm surrounding the plates. The slides were aged 2 to 5 days at room temperature (RT) or overnight at 70°C.

### Labeled probe preparation

Total genomic DNA (gDNA) from *Squalius pyrenaicus* and *Squalius alburnoides* nuclear non-hybrid males were extracted from muscle and fins using Phenol:Chloroform:Isoamyl alcohol (PCI) method (Sambrook et al. 1989). gDNA quality was assessed by agarose gel electrophoresis. Completely degraded DNA samples are not optimal to obtain a good labeled probe, even though the manufacturer’s protocol (Roche) suggests using fragmented DNA. The amount of gDNA was evaluated at first using NanoDrop 1000 (Fisher Scientific) by diluting the samples in double-distilled H_2_O 1:100, and subsequently using both NanoDrop and a QBit Fluorometer (Invitrogen). The probe was labeled with Dig-11-dUTP according to the manufacturer’s protocol (Roche cat N° 11745816910) with small modifications. The amount of starting DNA was increased to 1.3–1.5µg and the incubation time was extended to 135 - 150 min in order to obtain a greater amount of labeled probe. Quality and quantity were assessed. After precipitation and air-drying, the probe was re-suspended in 40µl of GISH mix, a solution composed of 50% Dextran Sulphate (Promega), 10% SDS, 2× SSC, 500 ng/µl sheared salmon sperm DNA, 50x Denhardt’s and double-distilled H_2_O (final concentration of probe DNA 25ng/µl) during the first experiments (Set I, see below). In a second set of experiments (Set II), the labeled probe was re-suspended in Hybridization Mix composed of 50% Ultra Pure Formamide (Sigma F9037), 2× SSC, 10% Dextran Sulphate, and Milli-Q water; pH adjusted to 7–7.5 with 1N HCl. The initial probe DNA concentration was 20 ng/µl, but was later increased up to 200 ng/µl.

### Blocking DNA preparation

Genomic DNA for unlabeled blocking DNA was extracted from muscle and fins of non-hybrid samples (both AA and PP) using PCI method as described above. Several individuals were used in order to obtain a large amount of gDNA. After air-drying, the DNA was re-suspended in double-distilled H_2_O and the gDNA quality assessed by agarose gel electrophoresis. In this case it is not important if it is partially degraded. The DNA was then precipitated, air-dried and re-suspended in double-distilled H_2_O, vortexed and accumulated in a single tube. A hole was punched in the tube lid, and the suspension was autoclaved for 40 min. After autoclaving, an agarose gel electrophoresis test was performed to evaluate the rate of DNA shearing. The optimal size fragments for an efficient blocking DNA ranges from 100bp to 1000bp. Sheared DNA was then concentrated using a speed vacuum centrifuge (to about 5 µg/µl), and evaluated as described above.

### Genomic *In Situ* Hybridization (GISH)

Two sets of experiments (I and II) were performed.

Set I experiments (standard protocol)

The first experiments were performed according to the protocol used by Bi and Bogart (2006). Chromosomes were derived from old cell suspensions from diploid (PA) and triploid (PAA) specimens that were stored in fixative at -20ºC. Slide quality was evaluated as previously described and hybridization areas on the slide were identified using a diamond pen. The slides were aged for five days at RT. After aging, chromosomes were denatured for 2 min in Formamide 70%, 2× SSC at 72°C-74°C. The slides were then immediately dehydrated in a series of ice cold alcohols: 70% EtOH (7 min), 90% EtOH (7 min), 100% EtOH (10 min) and then air-dried.

Probe mix preparation

For each slide, the labeled hybridization probe mix was: 10µl Ultra-Pure Formamide (Sigma F9037), 5µl GISH mix, and 5µl Dig labeled probe (about 100ng). A range from 1:0 to 1:30 probe:blocking DNA (P/B ratio) was added to these solutions to optimize genomic differentiation. The solutions were stored at 4ºC prior to denaturation.

Probe denaturation and hybridization

During slide air-drying, the probe mixes were denatured for 7 min at 83°C in a PCR thermal cycler then immediately placed on ice for at least 10 min. 20 µl of probe mix was dropped onto each slide, a 22x22 mm coverslip was applied, sealed with parafilm, and incubated overnight at 37°C in a moist chamber.

Post-hybridization washes

Parafilm was removed and slides were immersed in 50% Formamide at 42°C for 10 min followed by three washes in 1× SSC for 5–7 min each at 42°C with gentle hand shaking. The washing steps were followed by rinsing in 2× SSC. To block unspecific binding sites in order to reduce fluorescence in the background, 70µl of Blocking Solution **(**BSA 5%, 20× SSC, double-distilled H_2_O and 0.1% Tween20) was applied to each slide, cover slips were then re-applied, and slides were incubated for 20 min at 37°C in a moist chamber.

Detection solution

Detection solution was made by diluting fluorescent antibody Anti-Digoxigenin FITC 1:100 in double-distilled H_2_O. 30µl of this solution was applied onto each slide, covered with a 22x22 mm coverslip and incubated for 80 min at 37°C in a moist chamber. The coverslip was then removed and the slide washed 4x 5 min in pre-warmed Washing Buffer (4× SSC, 0.1% Tween20) at 42°C. Then slides were rinsed in 2× SSC and counterstained using 1.5µg/ml Propidium Iodide (PI) or 1.5µg/ml DAPI (4’,6-diamidino-2-phenylindole) in antifade solution (Vectashield H-1300 and H-1200 respectively). Finally the slides were covered by a 24x32 mm coverslip and sealed with nail polish.

Microscope analysis

Slides were analyzed using a Leica DMRB fluorescence microscope. Fluorescent images from FITC, PI and DAPI were captured by a CCD camera (QImagine, Vancouver, Canada) and merged using Openlab 3.5 Software. All the images were analyzed and slightly manipulated with Adobe**®** Photoshop Elements 6 and Adobe**®** Photoshop CS4.

Set II experiments (GISH protocol optimization and validation)

Several parameters were modified in order to improve *in situ* hybridization quality, which is important for an accurate interpretation of GISH results. All the modifications were introduced step by step, and other experimental conditions remained constant. The old cell suspensions that provided chromosomes were initially used in parallel with the two new cell suspensions, and every modification was introduced as a fixed parameter in the following trials.

Water type

The first optimization experiments were performed using the same chromosome suspensions in the same conditions changing only the water type from di-deionized to Milli-Q Millipore**^®^** (18.2 MΩ·cm) in all the solutions.

Pepsin pretreatment (optional)

Pepsin pretreatment was performed before the denaturation but only on the slides in which metaphase spreads were surrounded by abundant cytoplasm. Slides were placed 10–12 min in pre-warmed (37°C) 0.01N HCl containing 0.002% pepsin (Sigma P7012). Pepsin activity was stopped by immersion in 1× PBS pH 7.4 for 5 min with gentle shaking, once in 2× SSC 5 min then dehydrated in a series of alcohol (70%, 90%, 100%) at RT.

Denaturation temperature

In order to preserve chromosomes morphology, denaturation temperature was decreased to 65°C and denaturation time extended from 2 minutes up to 3 minutes.

Post-hybridization washes

Post-hybridization washes were modified using 2 times 2× SSC, once 2× SSC+0.1% Tween20 and 1× PBST/0.5% w/v powder skimmed milk (as blocking solution). All these washes were performed for 5 min with gentle shaking at 42°C. In the following experiments these washes were performed at RT in order to reduce the stringency.

Probe re-suspension mixture

The labeled probe was re-suspended in Hybridization Mix composed of 50% Ultra-Pure Formamide (Sigma F9037), 2× SSC, 10% Dextran Sulphate (Promega), and Milli-Q water; pH adjusted to 7–7.5. The final concentration was 40–50ng/µl, and later increased up to 200 ng/µl.

Incubation times

The incubation time was progressively extended from overnight up to 5 days (see methods in Ali et al. 2004), though the best results were obtained with an incubation time of around 72 h.

Quantity of Probe

Initially, 100 ng of probe in a 22x22 mm hybridization area were used. Then the amount of the probe was increased to 200 ng, 500 ng, 1µg and a little bit more. Having suspensions containing many well-spread metaphases, the hybridization area was reduced to 10x10 mm, 12x12 mm or a 13 mm diameter. In these cases we applied from 200 to 350 ng of labeled probe on each slide.

P/B ratio evaluation

Probe/blocking DNA ratio was altered but used the same amount of labeled probe (1µg based on the previous tests) and the P/B ratio ranged from 1:20 to 1:40.

Pre-annealing test

In order to reduce the intensity of hybridization signals from shared highly repetitive sequences (e.g. rDNA, centromeric heterochromatin), a pre-annealing step of 30 min incubation at 37°C was introduced after probe denaturation (in a thermal cycler).

Microscope analysis

Slides were analyzed using an Olympus BX 60 fluorescence microscope. Fluorescent images from FITC, PI and DAPI staining were captured with an Olympus DP50 CCD camera. The images were analyzed and slightly manipulated with Adobe**®** Photoshop CS4.

## Results

**Figure 1.**
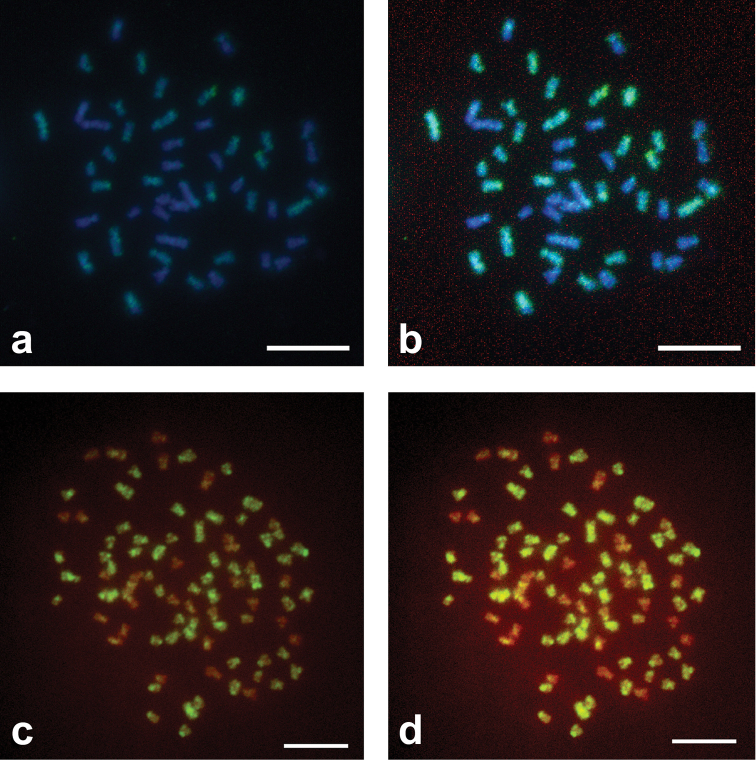
Standard use of GISH method in metaphase plates of specimens of *Squalius alburnoides* complex. **a** GISH with AA Dig labeled probe in PA metaphase plate revealed by anti-Dig FITC antibody counterstained with DAPI - picture without photo editing **b** the same picture with photo editing **c** GISH with AA Dig labeled probe in PAA metaphase plate revealed by anti-Dig FITC antibody counterstained with PI, picture without photo editing **d** the same picture with photo editing. Scale Bars = 10µm.

The first GISH experiments (Set I) on PA and PAA metaphase plates from old chromosome suspensions using the standard protocol of Bi and Bogart (2006), were made by applying 100 ng of Dig labeled AA whole genomic probe in presence of an excess of PP blocking DNA (P/B ratio from 1:0 to 1:30). Probe fluorescence was revealed by anti-digoxigenin FITC-conjugated antibody. The results showed non uniform, faint and spotted hybridization signals, mainly at pericentromeric chromosome regions and rDNA clusters as well as in some heterochromatic regions (Fig. 1a–d). For PA, only 5 or 6 chromosomes were uniformly stained and the fluorescence was faint (Fig. 1a and 1c). It was difficult to correctly identify all the A genome chromosomes in the PA diploid hybrid on the basis of centromeric signals. Also, in the PAA plates (Fig. 1c and 1d), hybridization signals were faint and it was more or less impossible to correctly identify or to distinguish A from P chromosomes. It was also not possible to evaluate P/B ratio differences because the results obtained when changing blocking DNA amount were more or less equivalent (not shown).

**Figure 2.**
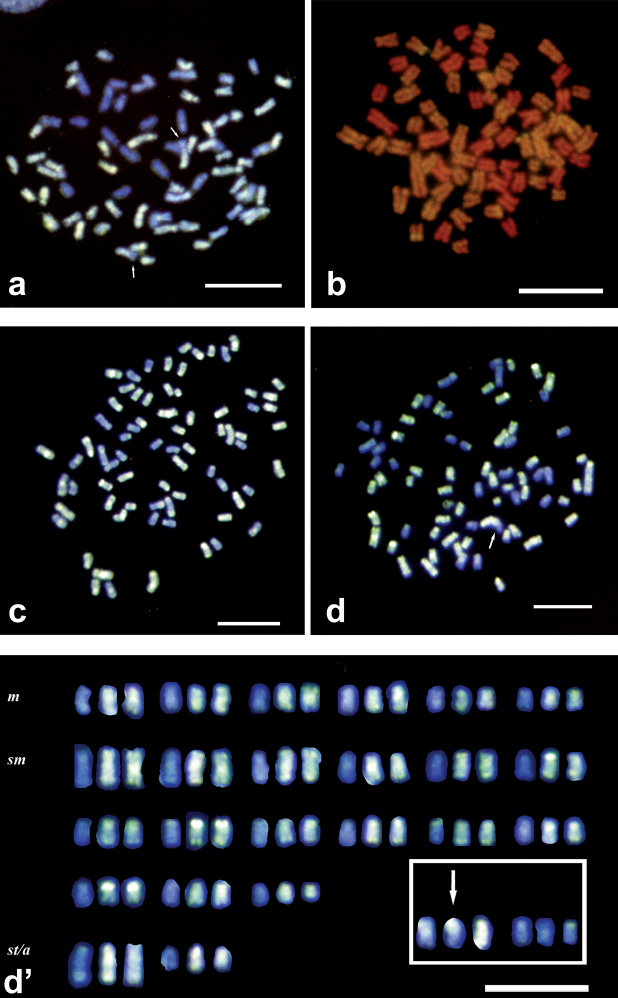
Improved use of GISH method and putative karyotype in specimens of *Squalius alburnoides* complex **a** GISH with AA Dig labeled probe in PAA metaphase plate revealed by anti-Dig FITC antibody counterstained with DAPI. Picture without photo editing. First improvement step **b-d** GISH with AA Dig labeled probe in PAA metaphase plates revealed by anti-Dig FITC antibody counterstained with DAPI (**c** and **d)** and PI (**b)**. Pictures with little photo editing. Last improvement step **d'** Putative PAA karyotype. Arrows indicate chromosomes putatively involved in exchanges, and box in karyotype some unresolved chromosomes. Scale Bars = 10µm.

The results from Set II experiments improved significantly by changing water type, post-hybridization washes, probe re-suspension mixture, pepsin pretreatment and fresh suspensions. MilliQ water contains fewer minerals affecting the hybridization of the probe. It stabilizes better hydrogen bonds between the probe and chromosomal DNA. Post-hybridization washes' modification reduced the stringency for the same reason. Pepsin pretreatment reduced fluorescent background, though no remarkable improvement was observed on hybridization quality. Reliable results were obtained when we used fresh chromosome suspensions, 65 h to 72 h incubation times and 1µg or more labeled probe per slide as shown in Figure 2a-2d. Uniform hybridization allowed to correctly identify parental chromosomes. Even when stronger signals occurred in rDNA clusters and at pericentromeric regions it was still possible to identify a few interchromosomal exchanges (Fig. 2d’). The optimal P/B ratio was 1:25. For suspensions containing a high number of metaphase plates, hybridization area reduction gave the same positive results (best size 12x12 mm or 13 mm diameter). This reduced the required amount of expensive labeled probe. In this set of experiments, little or no image editing was required to improve image quality.

## Discussion

When comparing the two sets of experiments and the improvements introduced to the protocol provided by Bi and Bogart (2006), it is evident that the metaphase chromosomes of the PA and PAA hybrids after GISH did not give reliable and informative results (Fig. 1a–d). Only the pericentromeric heterochromatin and some chromosomes of one of the two parental genomes could be identified but without absolute certainty. In most of the diploid and triploid hybrid plates a strong hybridization signal was observed at pericentromeric regions. Hybridization sites likely corresponded to heterochromatic areas showed by C-banding, which usually contain repetitive sequences, but it was not uniform and was often faint even in some centromeres. It was therefore impossible to clearly distinguish the two parental chromosomes sets. This problem was likely the result of a low concentration of the probe but also of the incorrect amount of blocking DNA.

Even though metaphase chromosomes used in Set I experiments appeared to be of very good quality and very clean when assessed by phase-contrast microscopy, the lack of genome specific fluorescence may be explained by the age of the cell suspensions as has been demonstrated both in plants and animals. Physical and chemical molecular modifications likely affect chromosome quality, and the use of fresh suspensions is always preferable (e.g. Henegariu et al. 2001).

Our experiments revealed other aspects that likely contributed to improve the quality of hybridization, such as those related to renaturation kinetics and the concentration of the probe. Hybridization kinetics that drives the formation of hetero- and/or homoduplex complexes depends on several factors: genome size, sequences copy number, DNA fragment size, base composition, concentration and time (Wetmur and Davidson 1968, Werman et al. 1996 and references therein). Salamanders of the genus *Ambystoma* have quite large genomes (22pg<GS>81pg, Gregory 2011). Coding sequences likely represent a very small fraction of the genome, which is mainly constituted by non-coding DNA including highly, moderate and interspersed repetitive sequences. Thus, in organisms with large genomes, reliable GISH results can be obtained with a short incubation time (overnight) and only a small amount of labeled probe is necessary (100ng or less). On the other hand, specimens from the *Squalius alburnoides* complex have a much lower C-value (about 1.2pg; Próspero and Collares-Pereira 2000). Consequently, considering renaturation kinetics theory, hybridization likely requires much higher probe concentrations (C_0_) and much longer incubation times (t).

Set II experiments increased probe concentration by reducing the hybridization area. A 22x22 mm area corresponds to 484 mm^2^. If 1µg of probe is used in this area it means that a Weight Specific Surface Area is about 2ng/mm^2^. Thus, operating on a reduced area, e.g. 100 mm^2^, it will be sufficient to drop only 200ng of the probe instead of 1µg. By adopting this system of hybridization area reduction it is possible to increase the concentration of the probe without using a higher amount of probe. The best results obtained with this procedure (Fig. 2b-2d)used 300ng of labeled probe in 4µl of Hybridization Mix in areas of 12x12 mm or 13 mm diameter (P/B ratio 1:25).

Improved protocol provided uniform and clean hybridization without any, or very little, requirement for image editing using more than 1µg of labeled probe and about 72 h of incubation in moist chamber at 37°C.

A few of the chromosomes contained mixed fluorescence along the arms (Fig. 2c–d), suggesting the occurrence of intergenomic exchanges (Rampin et al. 2010). However, though a tentative karyotype was built for distinct triploid (PAA) females, since parental chromosome sets are grossly very similar and constituted by a high number of relatively small chromosomes, we can not confirm the patterns of exchanges and identify the individual chromosomes that are involved (Fig. 2d’).

In vertebrates, intergenomic interactions have only been demonstrated by GISH in allopolyploid salamanders (Bi et al. 2007a, 2008, 2009, 2010). According to Zalesna et al. (2011) there were no such evidences in the hybridogenetic *Pelophylax esculentus* though only diploid hybrids were analysed. The results we have obtained confirm the power of GISH to define genomic composition in hybrids and to visualize interchromosomal exchanges (if present) even when dealing with hybrids of parental species having small chromosomes and C-values. Thus with the described improvements, GISH methodology will likely have a much wider application and be very helpful for cytogeneticists, when coupled with other molecular approaches, to unravel genome restructuring processes also in animal hybrid complexes.
